# Modelling perception-action coupling in the phenomenological experience of “hitting the wall” during long-distance running with exercise-induced muscle damage in highly trained runners

**DOI:** 10.1186/s40798-018-0144-1

**Published:** 2018-07-10

**Authors:** Andreas Venhorst, Dominic P. Micklewright, Timothy D. Noakes

**Affiliations:** 1 Department of Human Biology, Division of Exercise Science and Sports Medicine, University of Cape Town, Newlands, 7725 South Africa; 2 School of Sport, Rehabilitation and Exercise Sciences, University of Essex, Colchester, CO4 3SQ UK

**Keywords:** Structural equation modelling, Mediation analysis, Pacing behaviour, Perceived fatigability, Performance fatigability, Central regulation, Decision-making

## Abstract

**Background:**

“Hitting the wall” (HTW) can be understood as a psychophysiological stress process characterised by (A) discrete and poignant onset, (B) dynamic interplay between physiological, affective, motivational, cognitive, and behavioural systems, and (C) unintended alteration of pace and performance. A preceding companion article investigated the psychophysiological responses to 20-km self-paced treadmill time trials after producing exercise-induced muscle damage (EIMD) via a standardised muscle-lengthening contraction protocol.

**Methods:**

A 5-step procedure was applied determining the extent to which the observed data fit the hypothesised cause-effect relationships. Running with EIMD negatively impacts performance fatigability via (A) amplified physiological responses and a non-adaptive distress response and (B) deterioration in perceived fatigability: increase in perceived physical strain precedes decrease in valence, which in turn precedes increase in action crisis, eventually dissolving the initially aspired performance goal.

**Results:**

First, haematological indicators of EIMD predicted increased blood cortisol concentration, which in turn predicted increased performance fatigability. Second, perceived physical strain explained 44% of the relationship between haematological indicators of EIMD and valence, which in turn predicted increased action crisis, which in turn predicted increased performance fatigability. The observed data fitted the hypothesised dual-pathway model well with good model-fit indices throughout.

**Conclusions:**

The hypothesised interrelationships between physiological strain, perception, and heuristic and deliberative decision-making processes in self-regulated and goal-directed exercise behaviour were applied, tested, and confirmed: amplified physiological strain and non-adaptive distress response as well as strain-perception-thinking-action coupling impact performance fatigability. The findings provide novel insights into the psychophysiological processes that underpin the phenomenological experience of HTW and alteration in pacing behaviour and performance.

**Electronic supplementary material:**

The online version of this article (10.1186/s40798-018-0144-1) contains supplementary material, which is available to authorized users.

## Key points

- “Hitting the wall” (HTW) can be understood as a psychophysiological stress process characterised by (A) discrete and poignant onset, (B) dynamic interplay between physiological, affective, motivational, cognitive, and behavioural systems, and (C) unintended alteration of pace and performance.
- Running with EIMD causes (A) amplified physiological responses and a non-adaptive endocrinological distress response and (B) increase in perceived physical strain, which in turn mediates decrease in valence, which in turn predicts increase in action crisis; and both physiological and perceptual effects predict increase in performance fatigability.
- The findings provide novel insights into the psychophysiological processes that underpin the phenomenological experience of HTW under the constraint of amplified physical duress.
- Structural equation modelling and the applied three-dimensional framework of perceived fatigability comprehensively address pressing questions about how hypothesised cause-effect relationships come to be and how strain-perception-thinking-action coupling determines the observed alteration in pacing behaviour and endurance performance.

## Background

The “hitting the wall” (HTW) phenomenon is a much talked about but poorly understood phenomenological experience that regularly occurs in prolonged endurance exercise. HTW can be understood as a psychophysiological stress process characterised by (A) discrete and poignant onset and duration, (B) dynamic and complex interplay between physiological, affective, motivational, cognitive, and behavioural systems, and (C) unintended alteration of pacing behaviour and performance deterioration [1, 2].

The sheer physicality of endurance sports, particularly long-distance running, ensures that HTW is tightly coupled to stimulus-driven processes resulting from extreme physical duress. The most popular physiological explanation for HTW is skeletal muscle glycogen depletion and hypoglycaemia [3–5]. However, a computational study by Rapoport [6] showed that glycogen storage capacity is only a performance-limiting factor in runners with low and moderate aerobic capacities, but may not constrain performances of highly trained long-distance runners. An alternative determinant of HTW is exercise-induced muscle damage (EIMD) caused by muscular exertion of unaccustomed exercise duration and/or intensity, especially when involving muscle lengthening contractions [7]. This notion is supported by length of longest training run predicting the likelihood of HTW [1, 8] and correlations between deterioration in marathon running pace and indirect haematological markers of muscle damage [9].

The performance level-dependent nature of HTW [10, 11] points towards the importance of salient primary appraisals of physiological sensations. However, the large context-dependent intra- and inter-individual variability in subsequent psychophysiological responses necessitates the integration of sentient secondary appraisals of psychophysiological perceptions into a more holistic multi-dimensional framework [12].

Buman et al. [1] investigated the phenomenological characteristics of HTW in marathon runners and observed escalating generalised fatigue accompanied by unintentional slowing of running pace, increased desire to walk, and a shift from initially set performance goals to desire just finishing the race. Furthermore, runners experiencing HTW felt more negatively impacted by these events, and the authors interpreted the renegotiation of race aspirations as an adaptive behavioural response to exhaustion and performance deterioration as it did not per se increase the likelihood of race drop-out [1].

In a subsequent study, Buman et al. [2] qualitatively explored the psychological and behavioural experiences of HTW. Raw data were thematised into five higher-order themes: (A) affective (e.g. frustration), (B) behavioural (e.g. pace disruption), (C) cognitive (e.g. changing goals), (D) motivational (e.g. desire to quit), and (E) physiological (e.g. generalised and leg-related fatigue). As expected, physiological descriptors were reported most frequently [13, 14], but the authors concluded that the complex interactions of physiological, affective, motivational, cognitive, and behavioural perceptions truly underpin the goal-disengagement impulse associated with the HTW phenomenon [2].

Goal-related cost-benefit thinking in goal-striving consistently peaks around the 32-km mark during a marathon before resolving closer to the finish line [5, 8, 15]. Thus, non-linear dynamics of a psychological crisis concord with linear dynamics of a physiological crisis during HTW, but can dissociate thereafter. More importantly, deliberating anew the desirability and feasibility of the initially aspired performance goal during a marathon independently predicted increased performance fatigability, thereby providing a psychobiological link between a mindset-shift in goal-striving and performance degradation [5, 16, 17].

However, the above studies relied on correlational study designs, recreational runners, and low temporal resolution in perceptual and performance measures. These studies therefore provide limited information on pressing questions about (A) how hypothesised cause-effect relationships determining endurance performance come to be [18] and (B) how perception-action coupling underpins self-regulation and decision-making in pacing behaviour and endurance performance [19].

A recently proposed three-dimensional framework of centrally regulated and goal-directed exercise behaviour [20] was therefore applied to investigate the alterations in pacing behaviour and endurance performance in highly trained long-distance runners during a self-paced 20-km treadmill time trial after inducing EIMD by means of a standardised drop-jump protocol [7]. The preceding companion article showed dynamic changes and interdependencies in physiological and perceptual effects on pacing behaviour and performance fatigability that are in full agreement with the defining characteristics of the phenomenological experience of HTW described previously. An accepted approach to lend credibility to the hypothesised cause-effect relationships is to statistically test the integrity of the hypothesised model structure in relation to observed data.

Structural equation modelling (SEM) is a causal inference method that combines testable causal hypotheses based on scientific knowledge and empirical data as input, and produces quantitative causal claims, conditional on the input assumptions, as output [21]. The statistical inference is based on a simultaneous analysis of the entire system of variables determining the extent to which the observed data fit the hypothesised a priori specifications [22]. It thereby provides a higher-level perspective to the analysis and evaluation of entire models. Another key component to theory development is mediation analysis, which aims to clarify how cause-effect relationships come to be, [23] and which are therefore theoretical formulations for unidirectional relationships [24]. Thus, specifically when used in conjunction with an experimental design, the capabilities to formalise and implement causal inference makes SEM an indispensable tool in causal hypothesis testing and mediation analysis; its potential for theory development is even greater [25].

Here, SEM was applied to test the extent to which the observed data fit the hypothesised cause-effect relationships of the dual-pathway model outlined below and which are described in detail in the preceding companion article [7]. Due to the limited sample size, a simplified version was tested that focused on the haematological indicators of EIMD and thus the muscle damage component rather than the combined effects of locomotor muscle fatigue and EIMD. Differential responses in haematological indicators of EIMD are hypothesised to precede (1) amplified physiological and non-adaptive endocrinological distress response (indicated by blood cortisol concentrations) and (2) increase in perceived physical strain, which in turn mediates a decrease in valence, which in turn precedes an increase in action crisis, and both physiological and perceptual effects precede performance fatigability.

## Methods

Twenty-two (11 females) highly trained (performance level 4) runners completed two maximal self-paced 20 km treadmill time trials over a simulated profiled course in a counterbalanced crossover design: (A) in a tapered condition and (B) with EIMD produced by a standardised drop-jump protocol. Indicators of muscle damage, muscle metabolic strain, and endocrinological stress were assessed to investigate the physiological effects, and a three-dimensional framework of perceived fatigability emphasising the distinct contributions of sensory, affective, and cognitive processes was applied to investigate the perceptual effects of running with EIMD on performance fatigability.

The methods and results of preliminary and experimental procedures as well as the outcome of conventional statistical analyses have been described in detail in the preceding companion article [7]. A 5-step procedure was applied to ascertain that the theorised relationships were statistically robust enough for the structural modelling process [17].

In step one, conventional statistical analyses and visual inspection of data took place. Main study variables that showed significant treatment × time interaction or main treatment effects were considered potential explanatory moderators and mediators of defended regulatory variables and observed trial-related differences in performance fatigability. To overcome discrepancies in temporal dynamics and sampling points as well as non-linearity in psychophysiological responses, trial-related differences in area under the curve^1^ (∆ AUC) were calculated for each main study variable before proceeding with correlation and regression analyses.

In step two, zero-order correlations and linear regressions were computed. Zero-order correlations were calculated between ∆ AUC in main study variables determining the strength of association between each variable pair. Significant correlations between variable pairs were followed-up with linear regression analyses, thereby allowing predictions to be made.

In step three, mediation analysis was conducted when a predictor variable was correlated to the proposed outcome variable and another dependent variable. Mediation analysis measures the significance of the indirect effect and the extent to which the mediator variable accounts for the relationship between the predictor and the outcome variable. Mediation analysis is particularly appropriate to use when (A) the predictor variable is experimentally manipulated and the mediator variable measured and (B) the dynamic change in the predictor variable precedes the dynamic change in the mediator variable; and both precede the outcome variable [23, 26].

In step four, multiple hierarchical regression analyses were conducted between pairs of study variables hypothesised to be linked in a temporal order and separate for each pathway hypothesised to impact performance fatigability^2^: Path one: (A) blood leucocyte count and blood cortisol concentration, (B) blood neutrophil count and blood cortisol concentration, and (C) blood cortisol concentration and performance fatigability. Path two: (D) blood leucocyte count and perceived physical strain, (E) blood neutrophil count and perceived physical strain, (F) perceived physical strain and valence, (G) valence and action crisis, and (H) action crisis and performance fatigability. Every variable pair was analysed by means of stepwise longitudinal regression analysis to ensure that the relationship between the direct predictor and outcome variable in each pairwise comparison was independent of descriptor and training variables as well as conventional predictors of endurance performance. Age and weight were entered in step one. Weekly mileage and volume of other aerobic training were entered in step two. VO_2peak_ relative to body mass and running economy (i.e. energy cost of running) were entered in step three. Lastly, the direct predictor of the outcome variable of interest was entered in step four.

In step five, main study variables were fitted into a singular structural path model. Despite the small sample size (*n* = 22), SEM was deemed appropriate as the variables included in the tested model: (A) showed significant interaction or main treatment effects in conventional statistical analyses, (B) showed significant zero-order correlations, (C) showed inter-individual homogeneity in responses, (D) showed trial-dependent changes that were in full agreement with the theoretical predictions, and lastly (E) as a result of the relatively low complexity level of the tested model [27].

### Statistical analysis

All conventional statistical analyses were conducted with IBM® SPSS® (Version 24.0, Chicago, IL, USA). Slopes and AUC were calculated using GRAPHPAD PRISM® (Version 6.00 for Windows, GraphPad Software, La Jolla, CA, USA). Mediation analysis and structural equation modelling was performed using IBM® SPSS® Amos (Version 24.0, Chicago, IL, USA). Model fits were estimated using the maximum likelihood method, and results are represented as standardised estimates. Significance of the indirect effect within mediation analysis was estimated by means of bootstrap sampling and bias-corrected 95% confidence intervals based on 2.000 bootstrap samples [28]. Statistical significance was accepted at *p* < 0.05 (2-tailed).

## Results

### Step 1: Visual inspection and conventional statistical analyses

**Fig. 1:**
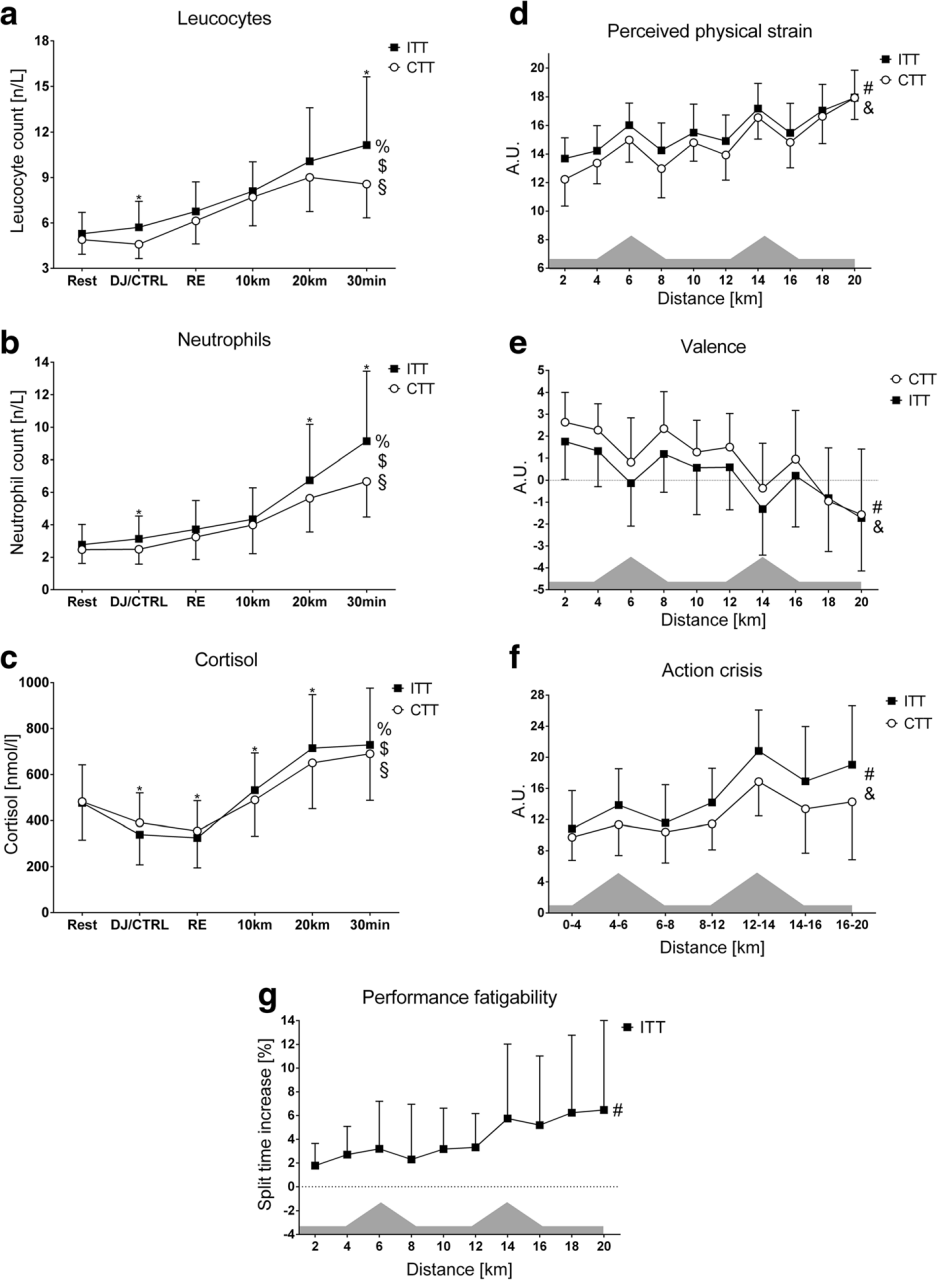
Trial-related differences in main study variables in response to running with mild exercise-induced muscle damage. Panels show: **a** = blood leucocyte count; **b** = blood neutrophil count; **c** = blood cortisol concentration; **d** = perceived physical strain; **e** = valence; **f** = action crisis; **g** = performance fatigability Note the differences in *x*-axes of action crisis and haematological variables due to different sampling times. Abbreviations: % = treatment × time interaction effect; $ = simple (main) time effect for intervention trials; § = simple (main) time effect for control trials; * = simple (main) treatment effect; & = main treatment effect; # = main time effect; DJ/CTRL = drop-jump protocol versus control; RE = running economy test; CTT = control time trial; ITT = intervention time trial

Trial-related differences in main study variables showing significant treatment × time interaction or main treatment effects are shown in Fig. 1.

### Step 2: Correlation and linear regression

Zero-order correlations among the major study variables are provided in Table 1.

**Table 1:** Zero-order correlations between trial-related differences in area under the curve of main study variables

	1	2	3	4	5	6
1. Δ AUC-leucocytes	–					
2. Δ AUC-neutrophils	.98**	–				
3. Δ AUC-cortisol	.56**	.59**	–			
4. Δ AUC-perceived physical strain	.54*	.52*	.33	–		
5. Δ AUC-valence	− .65**	− .69**	− .58**	− .78**	–	
6. Δ AUC-action crisis	.45*	.47*	.30	.40	− .51*	–
7. Performance fatigability	.36	.39	.51*	.12	− .36	.57**

Abbreviations: *Δ AUC* = trial-related difference in area under the curve. **Correlation is significant at the 0.01 level (2-tailed). *Correlation is significant at the 0.05 level (2-tailed)

Significant linear relationships were found between haematological indicators of EIMD (i.e. ∆ AUC in blood leucocyte and neutrophil count) and ∆ AUC in blood cortisol concentration, which in turn showed a significant linear relationship with performance fatigability (β = 0.51; *p* = 0.015). However, ∆ AUC in haematological indicators of EIMD also showed significant linear relationships with ∆ AUC in perceived physical strain, valence, and action crisis (although only weakly with the latter). Furthermore, there were significant linear relationships in the hypothesised temporal order between the ∆ AUC in perceived physical strain and valence (β = − 0.78; p = < 0.001), valence and action crisis (β = − 0.51; *p* = 0.015), and action crisis and performance fatigability (β = 0.57; *p* = 0.006). The simultaneous significant linear relationships between ∆ AUC in haematological indicators of EIMD, perceived physical strain, and valence required the examination of a potential indirect effect of perceived physical strain on the relationship between haematological indicators of EIMD and valence.

### Step 3: Mediation analysis

**Fig. 2:**
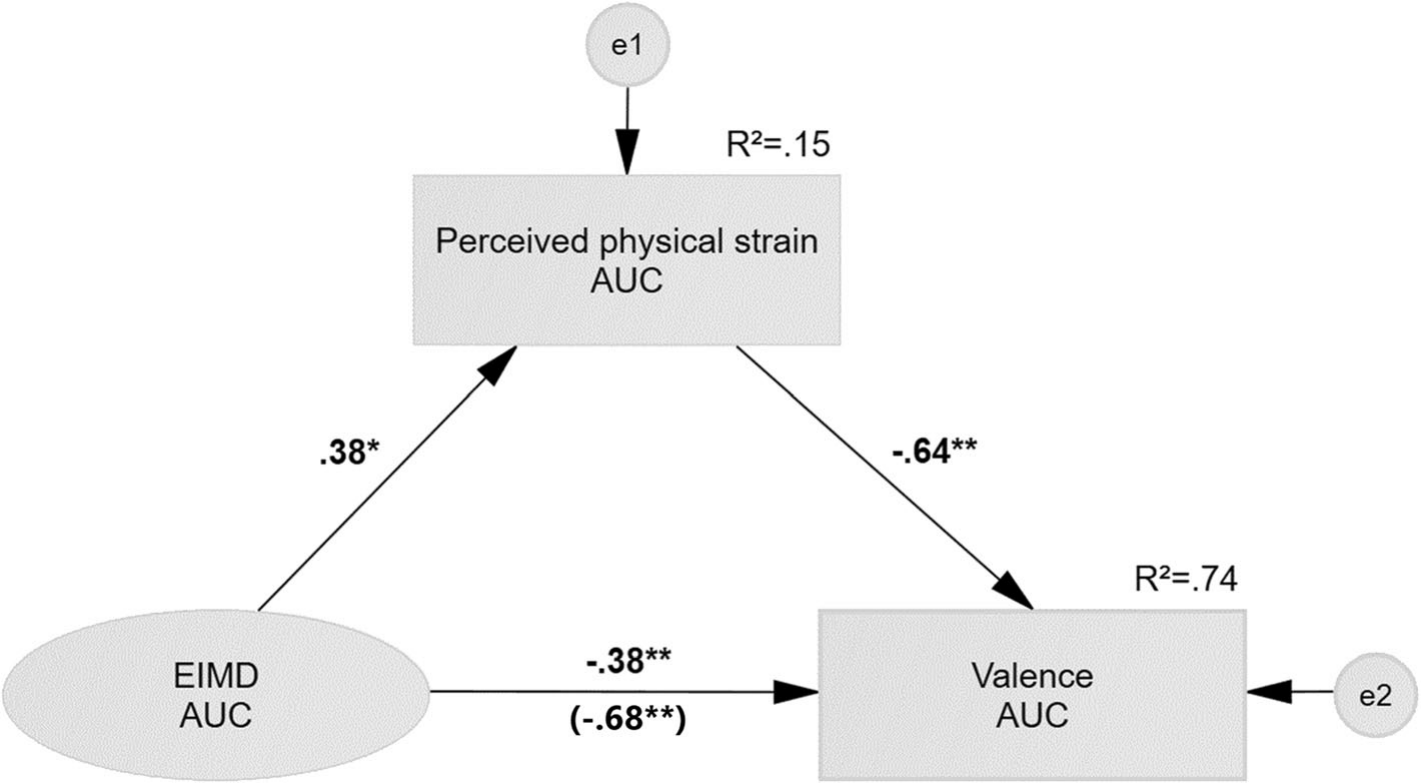
Mediation model of haematological indicators of exercise-induced muscle damage (EIMD), perceived physical strain, and valence. Note: trial-related differences in area under the curve (∆ AUC) of blood leucocyte and neutrophil count were used to indicate greater extent of EIMD during intervention trials. For graphical simplicity, indicators and errors are not shown. ∆ AUC of perceived physical strain was a significant mediating factor in the relationship between ∆ AUC in haematological indicators of EIMD and valence. Abbreviations: squares represent observed variables; ovals represent latent variables; single-headed arrows represent regression paths; bold regression paths are significant at *p < 0.05 and **p < 0.01, respectively; EIMD = exercise-induced muscle damage; AUC = area under the curve; *R*^2^ = total variance explained; *e* = residual error

In full agreement with our theory-driven approach, ∆ AUC in perceived physical strain was a significant mediator as it explained 44% of the relationship between ∆ AUC in haematological indicators of EIMD and valence. The significant total effect c (standardised estimate = − 0.68**, *p* < 0.001) between ∆ AUC in haematological indicators of EIMD and valence was reduced by a non-trivial amount to a significant direct effect *c’* (standardised estimate = − 0.38**, *p* = 0.001) after controlling for the significant indirect effect *a* × *b* (standardised estimate = − 0.24, *p* = 0.025). Statistical details are provided in Fig. 2 and Table 2.

**Table 2:** Mediation analysis of perceived physical strain accounting for relationship between exercise-induced muscle damage and valence

Effect	Standardised estimate	CR	SE	95%CI	*p*
Path *c* (total effect)	− .68	− 4.16			< .001
Path *a*	.38	2.09			.036
Path *b*	− .64	− 5.37			< .001
*a* × *b* (indirect effect)	−. 24		.145	[− .022; − .563]	.025
Path *c*’ (direct effect)	− .38	− 3.22			.001

Note: Maximum likelihood estimates are provided for paths *c*, *a*, *b*, and *c*’. For the standardised (*a* × *b*) indirect effect, bootstrap estimates with CIs are provided. Abbreviations: *CR* = critical ratio; *CI* = confidence interval; *SE* = standard error

### Step 4: Multiple hierarchical regression analyses

None of the control variables (i.e. age, weight, weekly mileage, other training, VO_2peak_, and energy cost of running) significantly predicted any of the main study outcome variables (i.e. blood cortisol concentration, perceived physical strain, valence, action crisis, and performance fatigability). More importantly, all direct predictors (i.e. blood leucocyte count, blood neutrophil count, blood cortisol concentration, perceived physical strain, valence, and action crisis) continued to significantly predict the respective outcome variable (i.e. blood cortisol concentration, perceived physical strain, valence, action crisis, and performance fatigability) after controlling for anthropometric and training variables as well as conventional predictors of endurance performance. The results of the seven stepwise regression analyses are summarised in Table 3.

**Table 3:** Multiple hierarchical regression analyses of control variables, direct predictor variables, and main study outcome variables

	Leucocytes ➭ Cortisol		Neutrophils ➭ Cortisol		Cortisol ➭ Performance fatigability		Leucocytes ➭ Perceived physical strain		Neutrophils ➭ Perceived physical strain		Perceived physical strain ➭ Valence		Valence ➭ Action crisis		Action crisis ➭ Performance fatigability	
Predictor	∆*R*^2^	β	∆*R*^2^	β	∆*R*^2^	β	∆*R*^2^	β	∆*R*^2^	β	∆*R*^2^	β	∆R^2^	β	∆*R*^2^	β
Step 1	.056		.056		.002		.066		.066		.022		.081		.002	
Age		− .130		− .130		.002		− .255		− .255		.035		− .183		.002
Weight		.201		.201		.042		.036		.036		− .144		.211		.042
Step 2	.196		.196		.012		.011		.011		.005		.094		.012	
Weekly mileage		.172		.172		.150		.058		.058		.029		−.424		.150
Other training		.538		.538		.124		− .063		− .063		− .051		.161		.124
Step 3	.004		.004		.047		.220		.220		.148		.015		.047	
VO_2peak_		− .069		− .069		− .182		.463		.463		− .175		.106		− .182
Economy		.067		.067		.244		− .514		− .514		.438		− .140		.244
Step 4	.372**		.395**		.291*		.307**		.276**		.528**		.210*		.461**	
Direct predictor		.708**		.726**		.626*		.643**		.608**		− .866**		− .504*		.755**

**Significant at the 0.01 level (2-tailed). *Significant at the 0.05 level (2-tailed)

### Step 5: Structural equation modelling

**Fig. 3:**
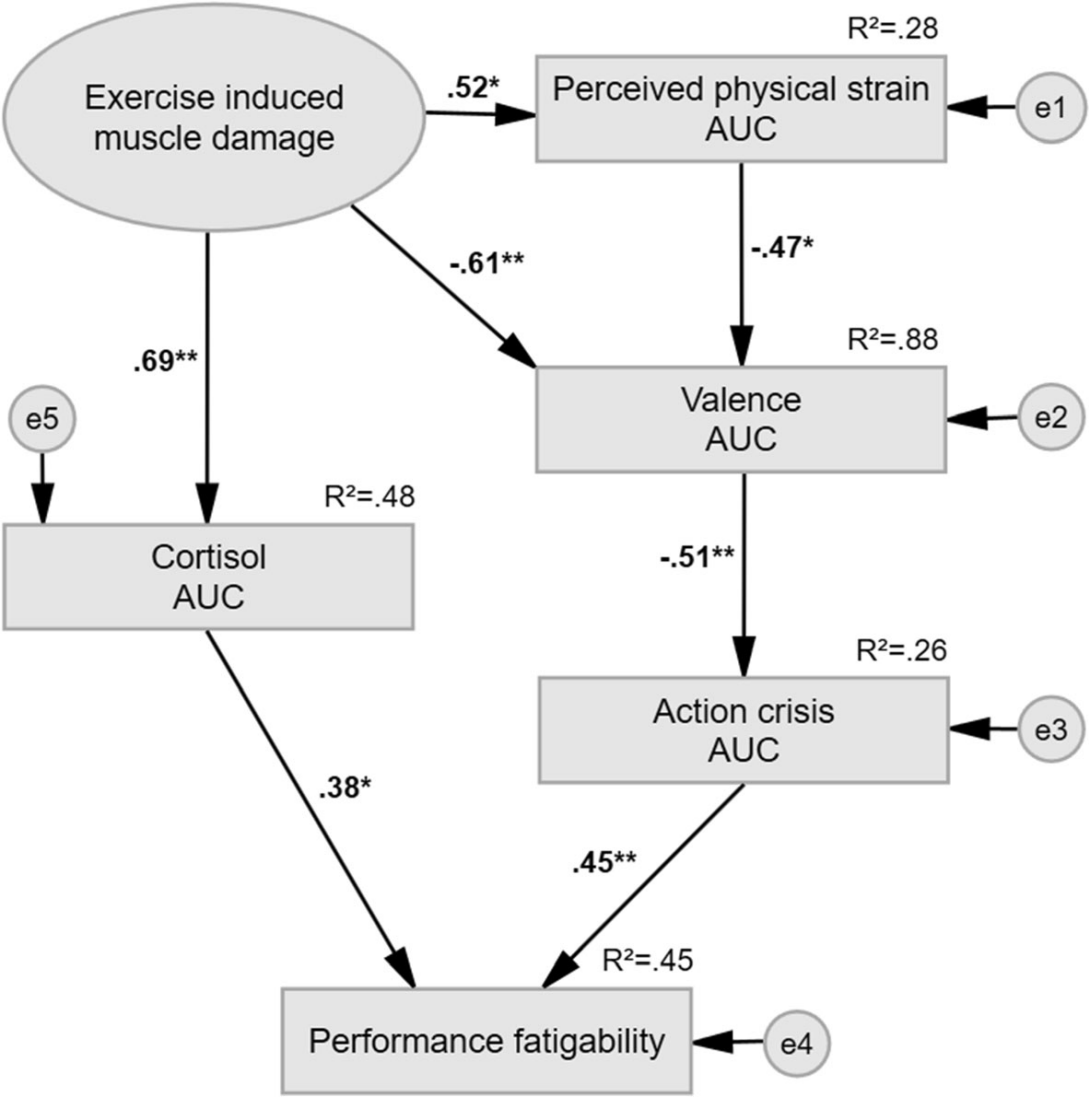
Structural equation model of physiological and perceptual effects on performance fatigability. Note: trial-related differences in area under the curve of blood leucocyte and neutrophil count were used to indicate greater extent of EIMD during intervention trials. For graphical simplicity indicators and errors are not shown. Abbreviations: squares represent observed variables; ovals are latent variables; single-headed arrows represent regression paths; bold regression paths are significant at *p < 0.05 and **p < 0.01, respectively; AUC = area under the curve; *R*^2^ = total variance explained; *e* = residual error; standardised maximum likelihood measures are used. χ^2^ = chi-square; NFI = normed fit index; CFI = comparative fit index; RMSEA = root mean square error of approximation; PCLOSE = p of close fit; AIC = Akaike information criterion; SRMR = standardised root mean square residual. Model fit indices are: χ^2^ = 7.277, *p* = .776, χ^2^/11 = .662, NFI = .949, CFI = 1.000, RMSEA = .000 (95%CI = [.000, .156]; PCLOSE = .806), AIC = 55.277, SRMR = .068

Details of the full structural equation model are provided in Fig. 3. The dual-pathway model hypothesised to underpin increased performance fatigability in response to running with EIMD entails: (1) debilitative physiological effects characterised by amplified physiological demand and non-adaptive endocrinological distress response and (2) debilitative perceptual effects characterised by deterioration in sensory, affective, and cognitive processes hypothesised to underpin perceived fatigability.

First, an increase in ∆ AUC in haematological indicators of EIMD significantly predicted non-adaptive endocrinological distress response indicated by an excessive increase in ∆ AUC in blood cortisol concentrations (standardised estimate = 0.69**; *p* < 0.01) despite reduced performance, which in turn significantly predicted increased performance fatigability (standardised estimate = 0.38*; *p* < 0.05).

Second, besides the significant mediatory role of perceived physical strain in the relationship between haematological indicators of EIMD and valence discussed previously, an increase in ∆ AUC in haematological indicators of EIMD significantly predicted an increase in ∆ AUC in perceived physical strain (standardised estimate = 0.52*; *p* < 0.05). An increase in ∆ AUC in perceived physical strain significantly predicted a decrease in ∆ AUC in valence (standardised estimate = − 0.47*; *p* < 0.05). A decrease in ∆ AUC in valence significantly predicted an increase in ∆ AUC in perceived action crisis (standardised estimate = − 0.51**; *p* < 0.01), and an increase in ∆ AUC in perceived action crisis significantly predicted an increase in performance fatigability (standardised estimate = 0.46**; *p* < 0.01).

The observed data fitted the hypothesised dual-pathway model well with good model fit indices throughout and a 78% probability that the observed data fit the hypothesised cause-effect relationships: χ^2^ = 7.277, *p* = .776, χ^2^/11 = .662, NFI = .949, CFI = 1.000, RMSEA = .000 (95%CI = [.000, .156]; PCLOSE = .806), AIC = 55.277, SRMR = .068) [29].

## Discussion

The present research applied SEM to measure the extent to which the observed data of the preceding companion article [7] fit the previously outlined psychophysiological cause-effect relationships hypothesised to shape the HTW phenomenon by combining causal (i.e. formalising and implementing testable causal hypotheses in an experimental design) and statistical (i.e. assessing the extent to which the experimental data fitted the hypothesised model) inference. Good model fit indices confirmed the credibility of the hypothesised model structure. The main findings were (A) discrete and poignant event onset, (B) differential responses in haematological indicators of amplified muscular strain and non-adaptive endocrinological distress response, (C) sensory-discriminatory, affective-motivational, and cognitive-evaluative processes indicative of deterioration in perceived fatigability, and (D) unintended alteration of pacing behaviour and performance fatigability.

First, the muscle lengthening contraction protocol caused a large 11% decrease in power output generating capacity of the knee extensors, medium increases in haematological indicators of EIMD, and large increases in symptoms of delayed onset of muscular soreness in highly trained runners; collectively confirming mild EIMD (for details see preceding companion article) [7]. Still, strength loss was only about half of that observed in similar studies using moderately trained cyclists [30, 31]. Thus, given that highly trained runners are arguably better adapted to mechanical strain through the repeat-bout effect, this might explain why the current study only observed trends towards significant zero-order correlations between haematological indicators of EIMD and performance fatigability.

However, the drop-jump protocol did induce medium increases in absolute cardiovascular, respiratory, and metabolic demands during the steady-state running economy test preceding the time trials, thereby indicating an increase in relative exercise intensity (for details, see preceding companion article) [7]. Running with mild EIMD therefore elicited a stressful physiological milieu that is not conducive to high-performance. In support, in the current study, differential responses in haematological indicators of EIMD predicted non-adaptive endocrinological distress response, which in turn predicted increased performance fatigability. Thus, the escalating leg-related and generalised fatigue experienced when HTW [2] are likely associated with greater accumulation of EIMD, amplified physiological demands, and excessive endocrinological distress response during the second-half of marathons compared to half-marathons [32]. This notion is consistent with observational data showing correlations between greater decreases in marathon running pace after ≈ 25 km and haematological markers of muscle damage in moderately trained runners who slowed down significantly more than those who were able to maintain their running speed [9].

Second, the most prevalent cognitions described by runners in response to challenging exercise [13, 14] are perceptions of salient physiological disturbance, and this particularly holds true under extreme physiological duress as experienced when HTW [2]. Another sentient characteristic of the phenomenological experience of HTW is that runners who HTW perceive these sensations to more negatively impact on their performance than those who do not, thereby becoming increasingly frustrated and discouraged [1, 2]. This attachment of valence to homeostatic disturbance and perceived physical strain eventually undermines effective goal-striving by promoting an urge to disengage from further goal-pursuit [33–35]. In support, in the current study, differential responses in haematological indicators of EIMD predicted an increase in perceived physical strain as well as a decrease in valence. Importantly, perceived physical strain was a significant mediator explaining 44% of the relationship between haematological indicators of EIMD and valence. Thus, in full agreement with recent proposals of pacing behaviour and performance regulation, [20, 36–38] stimulus-driven heuristic processes are hypothesised to motivate the desire to walk and quit,^3^ thereby necessitating the opposing recruitment of goal-driven volitional processes in continued goal-striving.

Third, runners HTW have been found to renegotiate their initially set performance aspirations and change their goals to just finishing the race [1, 2]. This closely resembles an intra-psychic conflict between further goal-pursuit and goal-disengagement resulting from negatively valenced events in goal-striving—the hallmark of an action crisis [15, 16]. During an action crisis, the cognitive orientation shifts from an implemental mindset facilitating volitional tasks such as goal-striving in the face of adversity to a deliberative mindset facilitating motivational tasks such as deliberating anew the desirability and feasibility of the focal and alternative goals [39, 40]. More specifically, the experience of such a mindset-shift during a marathon predicted and negatively impacted running performance independently from the detrimental effects of the linearly developing physiological crisis [5, 16]. In support, in the current study, a decrease in valence predicted an increase in action crisis, which in turn predicted an increase in performance fatigability, thereby suggesting heuristic and deliberative antecedents in the goal-disengagement process.

In the academic domain, using cross-lagged panel path analysis, Brandstätter and colleagues [16, 41] provided convincing evidence that an action crisis temporally antecedes devaluation of goal-desirability and deterioration in perceived goal-attainability. Similarly, recent neuroeconomic research found that in a fatigued state, and moderated by endurance capacity, anticipated costs of future efforts are escalated, while the attractiveness of anticipated rewards are discounted [42]. During psychophysiological crises, stimulus-driven reactive control therefore seems to undermine goal-driven proactive control and negatively impact on thinking-action coupling in the self-regulatory control of goal-striving [43]. However, according to Brick et al. [44], endurance athletes can monitor and control their thoughts and actions in self-regulated endurance performance through metacognitive processes, albeit at the speculated cost of accruing mental fatigue. Thus, the HTW phenomenon can be understood as psychophysiological stress process draining on fatigable and temporally limited physiological and psychological resources, eventually leading to the dissolution of the initially aspired performance goal.

Last, intriguing neurofunctional evidence for the above outlined hypothesised dual-pathway model has been provided by Maier et al. [45], who showed that acute stress biased decision-making and self-regulatory control via two parallel but distinguishable neuroanatomical pathways: (1) endocrinological distress (indicated by salivary cortisol concentration) increased connectivity with limbic system structures suggestive of augmented salient motivating stimuli (e.g. thereby facilitating the motivational desire to slow down) and (2) perceived stress levels decreased connectivity with prefrontal cortex structures suggestive of attenuated volitional goal-maintenance (e.g. thereby debilitating the volitional drive to attain the initially set performance goal).

### Limitations and future directions

First, a limitation of the current study is the sample size. The current findings therefore prevent the generalisation of findings beyond the specific context in which this study was conducted. However, the findings do lend credibility to the hypothesised cause-effect relationships and provide researchers with valuable information to drive further theory development and research design in strain-perception-thinking-action coupling during prolonged endurance exercise.

Second, to reduce data volume and simplify the modelling process, we focussed on action crisis to indicate the shift from an implemental to a deliberative mindset. However, the preceding companion article also showed mirror-like responses in flow state [7]. Thus, although model fit indices throughout were slightly better for action crisis compared to flow state (∆ AIC = 2.755), both measures seem valid indicators of a shift from an implemental mindset cognitively tuned towards the “how” of behaviour to a deliberative mindset cognitively tuned towards the “why” of behaviour (for statistical details see Additional file 1). This may provide the opportunity to also investigate facilitative effects of cognitive-evaluative processes in the regulation of pacing behaviour and endurance performance.

Third, given the significant zero-order correlation between blood cortisol concentration and valence, the fit of a theoretically equally feasible model with an additional unidirectional pathway from valence to blood cortisol concentration was explored. The alternative and more restrictive model showed slightly worse model fit indices (statistical results are not shown, but available from the first author upon request). Given the observed data, the initially hypothesised model is more parsimonious and therefore it is to be preferred based on this model comparison.

## Conclusions

By combining causal and statistical inference, the present article demonstrates that dynamic and complex interdependencies in physiological and perceptual effects determine the phenomenological experience of HTW and the behavioural outcome of observed pacing behaviour and performance fatigability during long-distance running with EIMD. More specifically, it contributes to a better understanding of how relationships come to be and how strain-perception-thinking-action coupling underpins observed pacing behaviour and performance fatigability. Differential responses in haematological indicators of EIMD predicted (1) amplified physiological strain and a non-adaptive endocrinological distress response and (2) increase in perceived physical strain, which in turn mediated a decrease in valence, which in turn predicted an increase in action crisis; and both physiological and perceptual effects predicted performance fatigability. The findings are in full agreement with recent theoretical developments in centrally regulated and goal-directed exercise behaviour and provide novel insights into the psychophysiological processes that (A) shape the phenomenological experience of HTW and (B) determine the poignant and unintended alterations in pacing behaviour and performance fatigability.

## Additional file

Additional file 1: Supplementary material contains graphical and tabular information on the 5-step structural equation modelling procedure using differential responses in flow state instead of action crisis: (1) trial-related differences in main study variables in response to running with exercise-induced muscle damage, (2) zero-order correlations between trial-related differences in area under the curve of main study variables, (3) multiple hierarchical regression analyses of control variables, direct predictor variables, and main study outcome variables, and (4) structural equation model of physiological and percpetual effects on performance fatigability. (PDF 452 kb)

## Abbreviations

∆ AUC: Trial-related differences in area under the curve
AIC: Akaike information criterion
CFI: Comparative fit index
CI: Confidence interval
CTRL: Control condition
CTT: Control time trial
DJ: Drop-jump protocol
EIMD: Exercise-induced muscle damage
HTW: “Hitting the wall”
ITT: Intervention time trial
NFI: Normed fit index
PCLOSE: p close of fit
RE: Running economy
RMSEA: Root mean square error of approximation
SEM: Structural equation modelling
VO_2peak_: Peak oxygen consumption
SRMR: Standardised root mean square residual.

## Footnotes

1 To reflect the lag period in haematological indicators of EIMD and endocrinological strain, areas under the curve of haematological variables were integrated from pre-time trial to post-recovery period.

2 The direct predictive effects of blood leucocyte and neutrophil count on blood cortisol concentration and perceived physical strain were assessed separately due to multi-collinearity.

3 Critically, the desire to walk and quit must be understood as a motivational drive as the vast majority of recreational runners HTW do not actually quit. [2] Similarly, the experience of an action crisis is not a binary event inevitably leading to self-regulatory failure, but constitutes an antecedent in the goal-disengagement process and does not necessarily imply that athletes let go of their goals entirely. [46]
