# Supplementary material for: Modelling perception-action coupling in the phenomenological experience of “hitting the wall” during long-distance running with exercise-induced muscle damage in highly trained runners
Source: Sports Med Open. 2018 Jul 10;4:30. doi: 10.1186/s40798-018-0144-1 (PMC6037658; doi:10.1186/s40798-018-0144-1)
Supplement: Supplementary file 1 — Supplementary material contains graphical and tabular information on the 5-step structural equation modelling procedure using differential responses in flow state instead of action crisis: (1) trial-related differences in main study variables in response to running with exercise-induced muscle damage, (2) zero-order correlations between trial-related differences in area under the curve of main study variables, (3) multiple hierarchical regression analyses of control variables, direct predictor variables, and main study outcome variables, and (4) structural equation model of physiological and percpetual effects on performance fatigability. (PDF 452 kb) [file 40798_2018_144_MOESM1_ESM.pdf]

SUPPLEMENTARY MATERIAL

Title:

Modelling perception-action coupling in the phenomenological experience of “hitting the wall” during long-distance running with exercise-induced muscle damage in highly trained runners

Journal:

Sports Medicine - Open

Corresponding author:

Andreas Venhorst; Department of Human Biology, University of Cape Town, Division of Exercise Science & Sports Medicine, Newlands 7725, South Africa; andreas.venhorst@gmail.com;

Co-author(s):

Dominic P. Micklewright; School of Sport, Rehabilitation and Exercise Sciences, University of Essex, Colchester UK. CO4 3SQ

Timothy D. Noakes; Department of Human Biology, University of Cape Town, Division of Exercise Science & Sports Medicine, Newlands 7725, South Africa

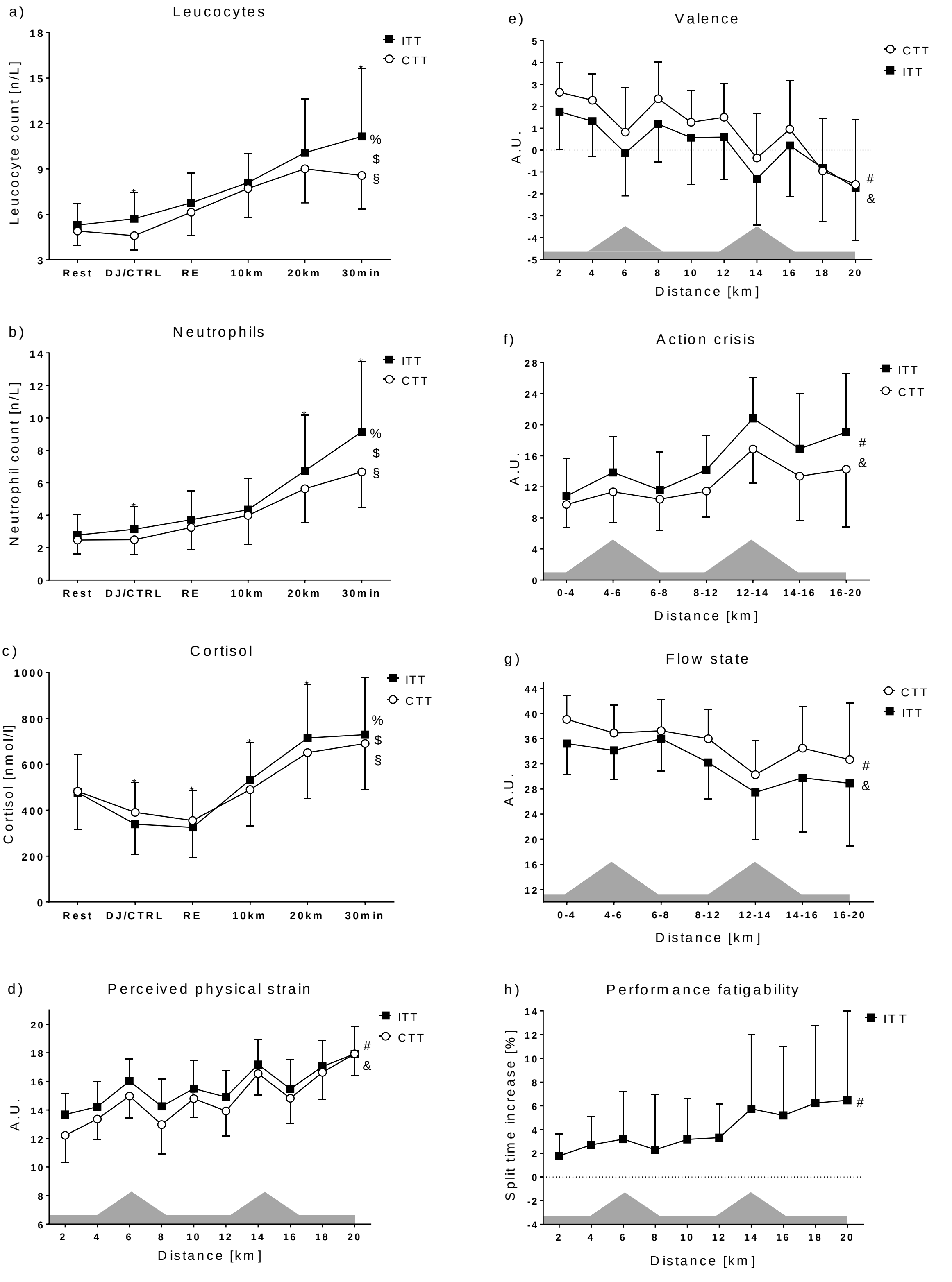

Figure SM 1 Trial-related differences in main study variables in response to running with exercise-induced muscle damage. Note the differences in x-axes of action crisis, flow state and haematological variables due to different sampling times.

Note: A.U. = arbitrary units; % = treatment × time interaction effect; $ = simple (main) time effect for intervention trials; § = simple (main) time effect for control trials; * = simple (main) treatment effect; & = main treatment effect; # = main time effect.

Table SM 1 Zero-order correlations between trial-related differences in area under the curve of main study variables.

	1	2	3	4	5	6	7
1. Δ AUC - Leucocytes	-						
2. Δ AUC - Neutrophils	.98**	-					
3. Δ AUC - Cortisol	.56**	.59**	-				
4. Δ AUC - Perceived physical strain	.54*	.52*	.33	-			
5. Δ AUC - Valence	-.65**	-.69**	-.58**	-.78**	-		
6. Δ AUC - Action crisis	.45*	.47*	.30	.40	-.51*	-	
7. Δ AUC - Flow state	-.41	-.42*	-.23	-.43*	.44*	-.74**	-
8. Performance fatigability	.36	.39	.51*	.12	-.36	.57**	-.58**

Abbreviations: Δ AUC=Trial-related difference in area under the curve. ** Correlation is significant at the 0.01 level (2-tailed). * Correlation is significant at the 0.05 level (2-tailed).

Table SM 2 Multiple hierarchical regression analyses of control variables, direct predictor variables, and main study outcome variables.

	Leucocytes ⇨ Cortisol		Neutrophils ⇨ Cortisol		Cortisol ⇨ Performance fatigability		Leucocytes ⇨ Perceived physical strain		Neutrophils ⇨ Perceived physical strain		Perceived physical strain ⇨ Valence		Valence ⇨ Flow state		Flow state ⇨ Performance fatigability	
Predictor	ΔR^2	β	ΔR^2	β	ΔR^2	β	ΔR^2	β	ΔR^2	β	ΔR^2	β	ΔR^2	β	ΔR^2	β
Step 1	.056		.056		.002		.066		.066		.022		.018		.002	
Age		-.130		-.130		.002		-.255		-.255		.035		.111		.002
Weight		.201		.201		.042		.036		.036		-.144		-.077		.042
Step 2	.196		.196		.012		.011		.011		.005		.020		.012	
Weekly mileage		.172		.172		.150		.058		.058		.029		-.148		.150
Other training		.538		.538		.124		-.063		-.063		-.051		.009		.124
Step 3	.004		.004		.047		.220		.220		.148		.003		.047	
VO_2peak		-.069		-.069		-.182		.463		.463		-.175		.028		-.182
Economy		.067		.067		.244		-.514		-.514		.438		.050		.244
Step 4	.372**		.395**		.291*		.307**		.276**		.528**		.209^		.346*	
Direct predictor		.708**		.726**		.626*		.643**		.608**		-.866**		.503^		-.601*

** Significant at the 0.01 level (2-tailed). * Significant at the 0.05 level (2-tailed). ^ Significant level = 0.068 (2-tailed).

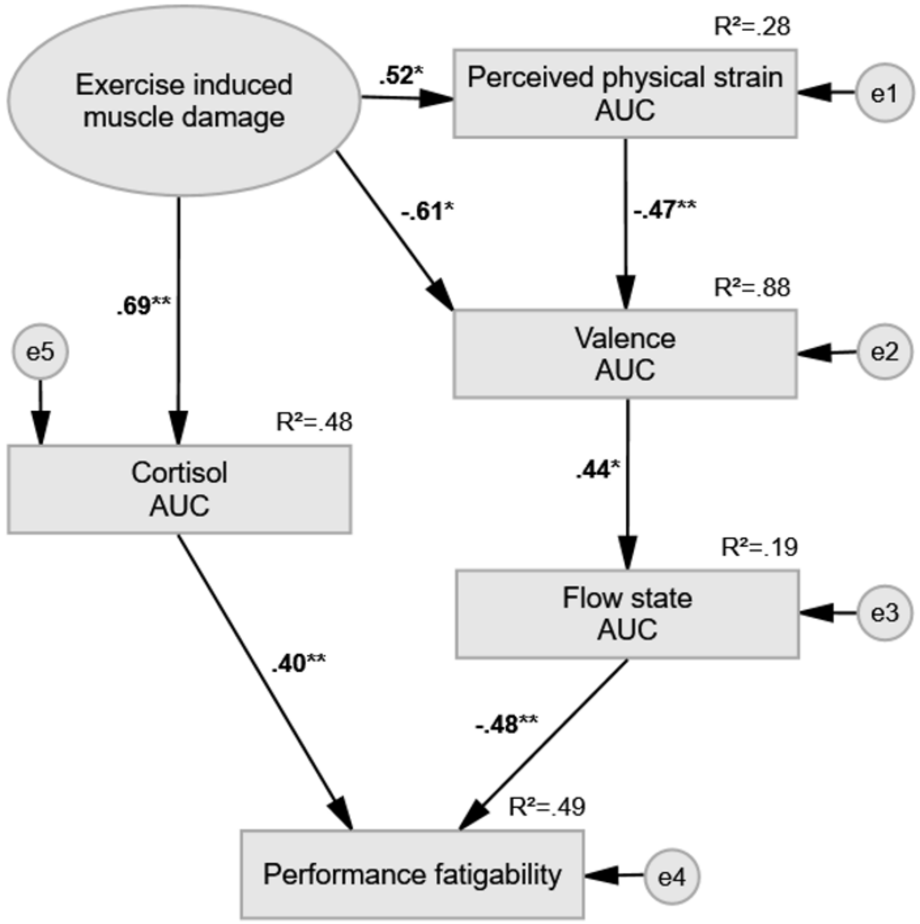

Figure SM 2 Structural equation model of physiological and perceptual effects on performance fatigability. Note: Trial-related differences in area under the curve of blood leucocyte and neutrophil count were used to indicate greater extent of exercise-induced muscle damage during intervention trials. For graphical simplicity indicators and errors are not shown.

Note: Squares represent observed variables; Ovals are latent variables; Single headed arrows represent regression paths; Bold regression paths are significant at * $p < 0.05$ and ** $p < 0.01$, respectively; AUC = area under the curve; R^2 = total variance explained; e = residual error; Standardized maximum likelihood measures are used. χ^2 = Chi-square; NFI = normed fit index; CFI = comparative fit index; RMSEA = root mean square error of approximation; PCLOSE = p of close fit; AIC = Akaike information criterion; SRMR = standardized root mean square residual.

Model fit indices are: χ^2 = 10.032, p = .528, χ^2/11 = .912, NFI = .931, CFI = 1.000, RMSEA = .000 (95%CI = [.000, .213]; PCLOSE = .573), AIC = 58.032, SRMR = .069.
